# Measurement of Community Empowerment in Three Community Programs in Rapla (Estonia)

**DOI:** 10.3390/ijerph8030799

**Published:** 2011-03-11

**Authors:** Anu Kasmel, Pernille Tanggaard Andersen

**Affiliations:** 1 Institute of Political Science and Governance, University of Tallinn, Narva mnt. 10-120, Tallinn, Estonia; 2 Institute of Public Health, University of Southern Denmark, Niels Bohrs Vej 9-10, 6700 Esbjerg, Denmark; E-Mail: ptandersen@health.sdu.dk

**Keywords:** health promotion, community empowerment, empowerment evaluation, Estonia, Eastern Europe

## Abstract

Community empowerment approaches have been proven to be powerful tools for solving local health problems. However, the methods for measuring empowerment in the community remain unclear and open to dispute. This study aims to describe how a context-specific community empowerment measurement tool was developed and changes made to three health promotion programs in Rapla, Estonia. An empowerment expansion model was compiled and applied to three existing programs: Safe Community, Drug/HIV Prevention and Elderly Quality of Life. The consensus workshop method was used to create the measurement tool and collect data on the Organizational Domains of Community Empowerment (ODCE). The study demonstrated considerable increases in the ODCE among the community workgroup, which was initiated by community members and the municipality’s decision-makers. The increase was within the workgroup, which had strong political and financial support on a national level but was not the community’s priority. The program was initiated and implemented by the local community members, and continuous development still occurred, though at a reduced pace. The use of the empowerment expansion model has proven to be an applicable, relevant, simple and inexpensive tool for the evaluation of community empowerment.

## 1. Introduction

Current health promotion policies and practices value community developmental projects that empower communities as a vehicle to achieve agreed upon health and social outcomes [1,2]. Empowerment is a principal theory in community psychology [3], and it is a key concept for communities aiming to achieve a better quality of life [2,4–6]. The Ottawa Charter identifies community empowerment as the core concept of health promotion discourse [7]. Indeed, a body of evidence exists in support of empowerment initiatives that lead to improved health outcomes and that represent viable health promotion strategies [2,8].

Empowerment approaches have been used for the prevention of non-communicable diseases in India [9], in suicide prevention among citizens of six towns in Japan [10], for avoidance of infant mortality in Boston, USA [11], for prevention of malaria in Thailand [12], and in many other initiatives. Although the concept of empowerment has met with wide acceptance in the scientific community and has proved successful in many Western countries [13], it is not confirmed, whether the same level of success will occur in the newly independent Eastern European countries is not demonstrated, as empowerment approaches in Eastern European countries, are still in their infancy as the socio-economic and socio-psychological prerequisites of long-term gradual improvement in the health status are weak and modest [14]. Only a few studies exist that highlight empowerment processes in countries in transition. The current study focuses on the empowerment issue in Estonia.

Empowerment is a complex issue. According to Zimmerman [15], empowerment may be viewed on different levels: individual, organizational or community. These levels are closely linked. In empowered communities, empowered organizations exist, and an empowered organization is reliant on the empowerment levels of its members. The current study focuses on community empowerment.

Community empowerment is understood either as a process or as an outcome [16–19]. Through the process of empowerment, communities are able to assume power to act effectively to change their lives and environment [20–22]. The community empowerment process promotes the participation of people, organizations and communities for increased individual and community control, political efficacy, improved quality of life and social justice [14]. The primary concept is to mobilize local communities to address their health and social needs and to work inter-sectorally to solve local problems [19]. Despite the wide use of empowering strategies in health and social development interventions [6] and by many researchers [2], there seems to be no consensus on agreed methods or universally accepted measurement tools to assist in the evaluation of the community empowerment process.

To evaluate the empowerment process, health promotion practitioners require a thorough operationalization of the community empowerment concept in health promotion interventions [23]. Furthermore, the paradigm of the evaluation approach and switch from a traditional hierarchical top-down approach to a participatory constructivist approach should be reconsidered [1,24–27].

The measurement of community empowerment relates to enabling community members to initiate and sustain activities leading to changes in the health and quality of life of the community. A range of factors and organizational aspects that affect empowering influence by community members have been suggested by Laverack and Wallerstein [19] and designated as the Organizational Domains of Community Empowerment (ODCE). The ODCE are connected to community members’ empowerment and own capacities to influence change. The term ODCE largely overlaps with the term community capacity domains; both describe factors that increase the assets upon which a community can variably draw on to improve their health, lives and well-being [28,29]. The community capacity concept, however, might not always include the community activation domain; it may also exist passively.

It is argued that ODCE offer a straightforward way in which to view, measure, and evaluate changes in community empowerment [19]. These are organizational domains that present an explicit lane to evaluate community empowerment as a process. To the best of our knowledge, no evaluation of the organizational domains of community empowerment has been undertaken in Estonia.

### 1.1. The Aim of the Study

The aim of this study is to follow up on the changes in ODCE after the application of the empowerment expansion model in the year 2003, one year later and two years later in three community initiatives in Rapla County, Estonia. The specific objectives were:

- to demonstrate the measurement process of the ODCE;
- to present the findings of the measurement of the ODCE; and
- to discuss the process, findings, limitations and implications of the study.

The paper is structured as follows: first, a description of the study context and settings is given; second, the concept of the ODCE is illustrated; third, the framework and methods are described; fourth, the findings are presented; and finally, the process, findings, limitations and implications are discussed.

### 1.2. Context and Settings

The current study was carried out in Rapla County, Estonia. Rapla County is a small inland region located in the western part of the country. It has an extension of some 2,980 km^2^ and about 37,400 inhabitants. It is a mainly rural area, with a small central town (Rapla, pop. 5,600). There are limited employment possibilities, and the relative poverty of the population in comparison to other regions in Estonia is high in comparison to the national average [30]. Rapla has a clearly defined geographical location; the people have a strong common identity and share common communication channels (local radio, newspaper).

In 1997, the Rapla County government appointed a health promotion practitioner. Since then, several health promotion efforts have been initiated, and other established nationwide health programs and projects were expanded into the county. Until the current study, previous assessments of health promotion initiatives were mainly focused on measuring health outcome changes. However, in 2002, the health promotion practitioner expressed the community’s desire to acquire information about empowerment approaches. In response to the community’s request, an empowerment evaluation study was designed to assess changes in empowerment.

The current study was implemented at three community health promotion initiatives in Rapla County: the Safe Community, Drug Abuse and AIDS prevention, and Elderly Quality of Life programs. The main differences between the involved programs concerned their approaches and orientation. The *Safe Community* program was initially a local *bottom-up* initiative, guided by the community workgroup, which involved interested people from different sectors—the non-governmental and private sectors—as well as some retired and unemployed persons. The workgroup was composed of community members who were aware of and concerned about the high rate of injuries occurring in the county, and who knew that there are options to improve the situation. The program was controlled by the community and focused on local capacity building. The program later involved municipal representatives and decision makers from different sectors. Large networks were formed involving more than 130 persons across the county. Hence, the program comprised a combination of a *top-down* and *bottom-up* initiatives. It practiced a combination of disease prevention methods through lifestyle management and community empowerment approaches. The program was financed on a yearly basis by a health promotion fund. It was a long-term collaboration and was initiated about five years before current evaluation.

The *Drug Abuse and AIDS Prevention* program was a more conventional, *top-down* program, which was initiated, planned and controlled by government bodies and had national goals, objectives and action plans. The program was guided by a local coalition, which consisted of representatives and stakeholders from different organizations and decision makers from the authorities, altogether about eighty people. The program mainly focused on lifestyle changes and practiced disease prevention strategies. The program was financed by the state budget for five years according to the national action plan.

*The Elderly Quality of Life* program was a *bottom-up* initiative, which consisted of elderly women, about forty people, who were interested in improving the life of elderly citizens in their community. The program’s main aim was to avoid the exclusion of older people, and to make efforts to keep them involved socially. Problems were defined by the community and the main focus of the program was community empowerment and social justice. Program was financed by the health promotion fund.

The empowerment expansion model (see below) was applied in all three of the community programs that this study examined.

### 1.3. Conceptualizing Organizational Domains of Community Empowerment

Several authors have constructed different but somewhat overlapping ODCE (Table 1). While working in two rural Fijian communities, Laverack [31] has identified nine ODCE: participation, leadership, problem assessment, organizational structures, resource mobilization, links to others, asking why, program management and the role of outside agents.

Smith *et al.* [32] found that the most referenced ODCE were: participation, knowledge, skills, resources, shared vision, sense of community and communication. Hawe *et al.* [33] identified a more general set of domains. The ODCE were comprised of three main activities: (i) building infrastructure to deliver health promotion programs; (ii) building partnerships and organizational environments which ensure sustainable programs and health gains; and (iii) building problem–solving capability. Bush *et al.* [34] elaborated on a *Community Capacity Index*, in which they distinguished four domains: network partnerships, knowledge transfer, problem solving and infrastructure development. Domains identified by Gibbon [35] and Bopp [36] overlapped almost entirely with those determined by the abovementioned researchers.

Researchers have suggested that community empowerment is a context and program-specific process [32,34,37]. This idea presumes that communities may be guided by general sets of organizational domains but that the interpretation of domains may differ in different communities [33]. Indeed, most authors admit that ODCE have not been tested in relevant settings and the context of different communities.

In Estonia, using qualitative interviews among community health promotion programs participants, four organizational domains of community empowerment (Table 2) were formed based on Rapla community members’ opinions and perceptions [38]:

- activation of the community;
- competence of the community in solving its own problems;
- program management skills and
- creating a supportive environment.

As the domains identified by the Rapla community largely overlapped those identified by Bush *et al.* [34], the *Community Capacity Index* framework was used as the basis for the development of the measurement tool. The questionnaire for the evaluation of the ODCE was elaborated using consensus workgroup methods.

The workgroup members were asked to express their perception and understanding of each domain and indicator, discuss them, and reach a consensus on characteristics. They agreed that there were a total of 36 indicators and 12 aggregated indicators, with nine measures for each domain. During the discussions, several statements describing indicators were redefined, specified and adjusted to the local context. This process reaffirmed the statements of Gibbon *et al.* [35], Laverack [30], Hawe *et al.* [33], Bush *et al.* [34], and Foster-Fishman [39] about the organizational domains of community empowerment are context specific.

### 1.4. Elaboration of the Measurement Tool

With the development of community empowerment, three levels of each domain were identified by community members. A similar number of levels were suggested by Bush *et al.* [34], but the content of the levels was predominantly context specific. The actual activities were recorded to show evidence that determined the ODCE by matching the activities against the indicators listed in the questionnaire. A ranking for each indicator, 1 (not at all/very limited), 2 (somewhat), 3 (substantial) and 4 (almost entirely/entirely), was agreed upon. The validation of a set of domains and indicators was tested by two other community workgroups. A fragment of the questionnaire is presented in the Table 3.

## 2. Methodology

### 2.1. Theoretical Framework

**Figure 1.**
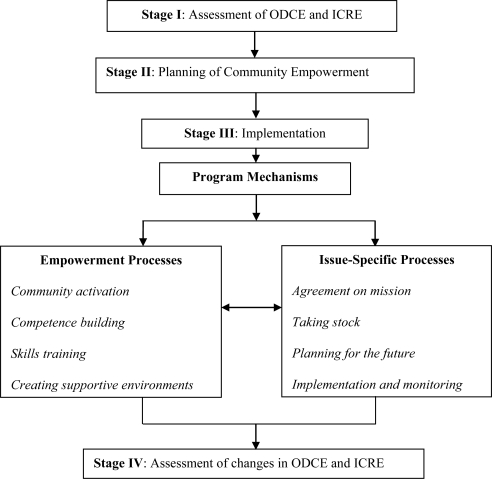
Empowerment expansion framework*. * Assessment of the individual community related empowerment (ICRE) is not presented in this paper.

The community workgroups constructed the empowerment expansion framework (Figure 1) to achieve and assess the changes in empowerment and health in three different programs that were being implemented. The framework was based on models of empowerment, as suggested by Fettermann [40], and the ‘parallel tracks’ program planning elaborated by Laverack [31].

Empowerment evaluation is defined as the use of concepts, techniques, and findings to foster improvement and self-determination [40]. It is an internal process by which participants themselves, in collaboration with health promotion practitioners, analyze their own program and work toward improving the quality of their program. Empowerment evaluation has an unambiguous value: to help people to help themselves using a form of self-evaluation and constant reflection. The advantage of the model is that it suggests to and teaches community members a simple, clear and convenient empowering guide. Its limitations are that it does not suggest how to conduct the empowerment concept, how to measure ODCE and how to evaluate changes.

According to the ‘parallel-tracking’ approach [31], community stakeholders create a separate set of goals and objectives for both issue-specific programs and community empowerment. The advantage of this model is that by clarifying and distinguishing two parallel processes in program development, participants focus not only on the ODCE but also the health issues, and they measure and evaluate the changes in both of the processes. In the current study, the assets of the above two models were combined.

The framework comprised four stages:

Stage I—assessment of ODCE (undertaken by the workgroups in the three community programs) and evaluation of the individual community related empowerment (ICRE). The latter is beyond the scope of the current paper and, hence, is not reported here.
Stage II—planning of community empowerment. This stage included the formulation and statement of the empowerment expansion, undertaken by workgroups at each of the three community programs, where goals and objectives for the empowerment expansion were defined, measurable indicators and measurement processes were identified, and action plans agreed upon.
Stage III—comprised two parallel implementation processes:
  - Empowerment expansion processes: these included numerous activities targeted on the development of the four ODCE domains (Table 2). These processes were debated on and formulated by the community that was being supported and facilitated and mediated by the health promotion practitioner and internal evaluator.
  - Issue-specific processes: in which the guidelines for empowerment evaluation [39] were used, and four actions were undertaken;
    - agreement on an issue-specific mission;
    - taking stock (activities undertaken so far were assessed, listed, analyzed, and rated, and an evaluation matrix was developed).
    - future planning (development of issue-specific goals and expected outcomes, and formulation of action plans). This step also included the selection of measurement tools, indicators and time-schedules for the issue-specific evaluation, *i.e.*, creation of a system of processes and outcomes monitoring; and,
    - implementation (including constant feedback and monitoring of issue-specific processes).
  In Table 4, some activities that were undertaken by community workgroups during the issue-specific processes are presented.
Stage IV—evaluation of changes in the ODCE (and assessment of the ICRE, which is not within the scope of the current paper). Thus, the current study assessed whether there were any changes in ODCE in three community programs workgroups during the implementation of the empowerment expansion framework in the three community health promotion initiatives.

The current evaluation was based on several assumptions. First, community groups were involved in each step of the evaluation, making all of the decisions through consensus building and sharing the ownership of the program. Second, community people themselves carried out the evaluation, whereby the evaluator and local health promoter acted as equal partners assisting, facilitating, enabling and mediating the process where needed. Third, the evaluation was undertaken in conformity with the local people’s needs and concerns.

The initial assessment was carried out in the beginning of the intervention in 2003 and thereafter followed up one and two years later, in 2004 and 2005.

### 2.2. The Process on the Measurement of the ODCE

For data collection, the consensus workshop method was selected. The method allows the identification and ranking of actual ODCE and collects examples of evidence to reconfirm it. The consensus workshop method is derived from a set of participatory group facilitation methods. The method encourages group member’s active participation and allows the use of information and ideas for the enhancement of the program [41].

The first workshops were carried out during the first empowerment evaluation planning meetings in January 2003 with three separate program workgroups. They followed up one and two years later, in January 2004 and 2005 respectively. Sixteen workgroup members in the Safe Community program participated in the workshop in the year 2003, twenty in the 2004 and seventeen in the 2005 (Table 5).

There were seven male and nine female participants in the Safe Community program in the year 2003, eight male and 12 female in 2004 and eight mail and nine female participants in the year 2005 ranging in age 29 to 68 years (mean age = 42.5 years in 2003, 44.8 in 2004 and 44.1 in 2005) with different backgrounds: medicine, social work, education, agriculture, economy, rescue system and two retired community members. Fourteen members of the Drug Abuse and AIDS Prevention program participated in the workshop in the years 2003 and 2004, and fifteen in the year 2005 (Table 5). Mean age of participants was ranging from 32.4 in 2003 to 36.1 in 2005. The workshop consisted of representatives of county government, local municipalities, schools, leisure centre, sport institution and health care system. Fifteen workgroup members in Elderly Quality of Life program participated in the workshop in the year 2003, eighteen in 2004 and seventeen in the year 2005. Twelve of participants were retired, three were working in education sector and two in health care sector.

The workshops started by setting the context. The facilitator outlined the process, topic, purpose and timeline for the workshop. The focus question, assessed by each domain separately, was introduced. Workshop participants were provided with the propositions of each indicator, asked to characterize a domain, and then asked to rank it using Likert-like measurement tool, from 1 (not at all/very limited), 2 (somewhat), 3 (substantial) or 4 (almost entirely/entirely). Every participant assessed the indicator individually at first. Rankings were then written on the board, and the group discussed them until a consensus was reached. The aggregation of the different levels of indicators were discussed, assessed and ranked thereafter, means and range of scores were calculated. After the proposition of each aggregated indicator, participants were asked to verify the evidence. At the end of the ranking procedure, the community workgroup discussed potential measures and opportunities to enhance each empowerment domain during the next program cycle. The next two evaluations of community capacity domains were carried out one and two years later, in January 2004 and 2005. They preceded the new empowerment evaluation planning cycles.

Ethical committee approval was not sought because in Estonia, studies that involve the voluntary participation of adults and have informed consent are exempt from further ethical approval.

## 3. Findings of the Measurement of the ODCE

Each indicator, aggregated indicator and level of indicators was determined after discussions and consensus among community members. For the purpose of visualization, rankings were calculated in numerical terms, and tables and graphs were developed for each initiative to include data from three measurements. The rankings used in the evaluation are not suitable for comparison of the three initiatives, but they do describe changes within each initiative over time. Furthermore, the evidences to describe changes were collected to illustrate and confirm the numerical findings.

### 3.1. Safe Community Program

Table 6 demonstrates that a remarkable increase in all four ODCE has taken place during the three year observation period. Domain levels demonstrate that the community has substantial ability to profit not only from local, but also from national and international knowledge and experience. The workgroup’s capacity to collaborate with partners on all levels has increased considerably. The data indicate that many new community members and influential leaders have joined the program. The Safe Community program network has expanded remarkably during three years and stakeholders maintained a commitment to the initiative. The most prominent change has occurred in the community competence domain—the awareness and knowledge on safety issues had increased remarkably. Significant change took place in the program management domain; workgroup members were able to collaborate as equal partners on national and international levels acquiring required skills and competencies to manage program implementation. Moreover, the indicators demonstrating capacities in building politically and financially supportive environments have increased substantially. At the end of the third year of measurement, the program had sustainable finances and support from decision-makers.

### 3.2. Drug Abuse and AIDS Prevention Program

The evaluation of the ODCE in the *Drug Abuse and AIDS Prevention* program demonstrated that capacities have substantially increased and were highest within the first three domains—community activation, community competence and program management (Table 7). The program during its first year made efforts to involve more stakeholders, among them young people directly endangered by the problem. Active leaders appeared among schools-children and youth organizations. Numbers of discussions were organized by the program members to raise the awareness and concerns and search solutions. Several training courses were implemented to improve management skills of stakeholders to be able to apply evidence based approaches. The fourth domain, the supportive environment, showed that political and financial support on the national and international level is easier to achieve if an issue is of national priority—the program was supported both by local and national decision-makers.

Although all of the ODCE were characterized by a steady and rapid increase, the discussions within consensus workgroups revealed that three years is a relatively short period for community development if the issue is not initially the local concern and that more time is needed for a community to create large networks and initiate external collaboration to prevent the newly appeared problem to expand.

### 3.3. Elderly Quality of Life Program

Results revealed that during the first study year program had a charismatic leader, who was able to mobilize new program members and motivate new leaders to take responsibility in community actions (Table 8). The program members gathered regularly to discuss issues concerned. Lots of events were organized and social life was activated. However, the program was unsuccessful in securing further financial support from the Health Fund. In second study year, when the program lost most of its finances, some organizational domains still increased, though more slowly. The workgroup was no longer as effective in mobilizing new groups and in recruiting new members into the program, but the activation domain still had slight increase. Although the competence development domain was perceived as being at a standstill, program management skills were increased through several training and the group was activated to focus to the application writing skills. However, the communication and collaboration with outside partners was limited and had slowed down. Despite the efforts in working with media and policy makers, the results were modest, and the capacity to influence policy makers and financers was assessed as weak.

## 4. Discussion

Community empowerment is said to offer the most promising approach for reducing health problems in communities [2,42]. Considering the remarkable gap and inequalities in health in between Western and Eastern European countries, the need for empowerment approaches in countries in transition is notable. Therefore, the empowerment expansion within the three health promotion community programs in Estonia in current study was perceived as a positive outcome.

Laferty [43] and Wallerstein [2] have argued that successful empowerment interventions cannot be fully shared or ‘standardized’ across multiple populations. Therefore, no one theory could be applied in its entirety to other populations but must be created within or adapted to local context. Also Smyth and Schorr [44] suggest that people must be seen in their real context. The current study has made efforts to consider these suggestions. The paper indicates the framework elaborated in cooperation with the community members for simultaneous empowerment and evaluation of the community process, combining the advantages of the empowerment evaluation [40] and the ‘parallel tracks’ [31] models.

Empowerment evaluation is a relatively new approach to evaluation. It has been adopted in higher education [45], community health promotion [46], violence prevention programs [47], in organizational changes [48] and in other areas, primarily in North America. Until now it has been applied modestly in Europe and to researchers’ knowledge not practiced in Estonia.

Although Fetterman *et al.* [40] have elaborated a simple and clear empowerment evaluation guide, they do not discuss the development of a practical methodology or “tools” for the measurement of community empowerment, nor do they assess whether the application of the model has resulted changes in community empowerment. This aspect has allowed opponents to criticize this approach. Patton [49] and Scriven [50], for example, have argued that Fetterman never demonstrated whether community members’ empowerment expanded as a result of the evaluation process. Nevertheless, the advantage of the model is that it suggests for communities a convenient empowering guide. Combination of empowerment evaluation model with the model developed by Laverack [31], which suggests a tool to measure ODCE in program development, was found a proper approach by the communities to evaluate the changes in empowerment expansion. The ODCE has presented an explicit lane to evaluate community empowerment as a process.

The framework was elaborated in collaboration with the community members and adapted to the local context by the community members during several discussions. The strengths of the framework and methodology used lied in the value-orientation - to help people to help themselves.

Crisp, Swerissen and Duckett [51] have argued that evaluation of the empowerment process is complicated because each community may identify and use a unique set of domains and empowerment strategies. The current approach confirms the argument as community identified and adopted the ODCE as they perceived it. The main strengths of the model were that it was developed, discussed and analyzed by the community and adapted to their context, so it was for community members understandable and easy to apply. The measurement of the ODCE was understood by the participants as an explicit and logical way to determine the required domains for the needed empowerment goals. Furthermore, the identification of existing domains assisted in the planning process of the empowerment expansion. The weakness of the current model is that it does not allow one to compare changes in empowerment expansion in different communities. However, more research is needed to identify the models’ compatibility for application in other communities.

Community health programs are initiated by local people in response to local needs (bottom up approach), by government requirements to solve national or municipal health problems (top to bottom approach) or by combined approaches. The evidence-based research has demonstrated that the most effective strategies are those that expand empowerment of local people and communities [2]. In current study in all three programs the ODCE were increased. However, evaluation of the programs indicated that the ODCE were increased most considerably among the community workgroups, which were initiated by community members and equally involved the municipality’s decision-makers—the *Safe Community* program. Local interest and initiative, the importance of the issue, and political, financial and expert support from decision-makers are crucial for community empowerment and further achievement of its goals. This argument is supported by Fawcet *et al.* [21] in his evaluation of community coalitions for the prevention of substance abuse.

The ODCE with the strongest political and financial support from the government institution was the *Drug Abuse and AIDS Prevention* program. The results demonstrated that the relevance of this issue among local people was critical. The dependence on a funding body and/or political requirements is important, but not enough to result in sustainable expansion of empowerment.

In the *Elderly Quality of Life* program the expansion of empowerment was relatively slow, but still evident. The community was unable to achieve any political or financial support from decision-makers, however, most empowerment domains, such as community activation, community competence, and program management skills still showed a steady increase. For the socially vulnerable groups, achieving both political and financial support was problematic. However, acquisition of social and expert support was attainable. Likewise, Crisp, Swerissen and Duckett [51] have found that it is difficult for program participants to achieve changes or develop without external and/or political assistance or support.

The consensus workshop method used for internal evaluation is a deceptively simple and yet powerful way to engage people and capture diverse ideas within community groups. According to Stanfield [41], consensus workshops promote inquiry; their intent is transformational. They allow people to respect and understand each person’s viewpoint and experience. Additionally, a consensus workshop method is transparent and serves and protects the interest and concerns of the group. The workshops’ inclusive consensus-building allows groups to have a high degree of consciousness in relation to the decisions it makes. Several researchers have emphasized the importance of this method in assessing community empowerment domains [29,34,39,52].

The findings of this study are limited by the fact that the workshops’ participants were not necessarily representative of all community members. According to Bopp [36], passive members are less likely to attend community development processes. In future research, these groups should be studied to fully understand the impact of empowerment strategies in larger groups in the community. The second limitation of this study is the small number of the participants, which limits the ability to generalize the findings. Finally, the results of this study are limited by the inclusion of participants who are proactive, as they joined the workgroups voluntarily. The extent to which these results reflect changes in ODCE on other groups is not known. There is a need for further research to clarify the results.

There are several implications of the study. Expansion of empowerment programs in communities is a powerful tool to help improve peoples’ health [53]. However, many health promotion practitioners have expressed their confusion concerning contradictions that exist between the essential nature of health promotion and the requirements of the politics, administrators and financiers that have evolved, primarily for traditional, medically oriented goals and objectives in community health promotion programs. The resources for health initiatives are mainly provided by the state budget and health promotion foundation for the predetermined initiatives, and usually, these are not in harmony with professionals’ understanding of effective approaches or local needs, concerns and interests. There is a need for a simultaneous empowering approach, its organization, and a pre-determined issue-specific approach. Furthermore, there is a need for the concurrent evaluation of both approaches. Health promotion practitioners, in collaboration with community members, can utilize the suggested approach to gain power and assess their own achievement in empowerment expansion. The implication of the current study is that it suggests to practitioners another possibility to measure the results of their health promotion program and gives another opportunity to be accountable. More financiers accept empowerment variables as targets that help health promotion practitioners focus directly on the main determinant of a community’s health status, the expansion of community empowerment and its organizational domains.

## 5. Conclusions

The use of the empowerment expansion model within different community programs demonstrated development of the ODCE in all three community health promotion programs. The current study suggests that, at least under some conditions, community program workgroups can empower themselves using contextually clarified ODCE and evaluating their implementation process. The community workgroup members agreed that this type of evaluation is a useful and flexible way of understanding and measuring the community empowerment process. It is also an applicable, rapid, simple and inexpensive tool that can be used in the measurement of the organizational domains of community empowerment. However, there is a need to test the same tool among more workgroups and communities.

## Figures and Tables

**Table 1.** ODCE by selected authors (adapted from [32]).

**Laverack [31]**	**Smith*et al.*[32]**	**Hawe *et al.*[33]**	**Bopp *et al*. [36]**	**Gibbon [35]**	**Bush *et al*. [34]**
- Participation - Leadership - Problem assessment - Organizational structures - Resource mobilization - Links to others - ‘Asking why’ - Program management - Role of outside agents	- Participation - Knowledge - Skills - Resources - Shared vision - Sense of community - Communication	- Building infrastructure to deliver health promotion programs - Partnerships and organizational environment - Problem solving capabilities	-Sense of community - Participation - Resources - Skills and knowledge - Leadership - Communication - Ongoing learning	- Representation - Leadership - Organization - Needs assessment - Resource availability - Implementation - Linkages - Management	*Community* *Capacity Index* - Network partnerships - Knowledge transfer - Problem solving - Infrastructure development

**Table 2.** Organizational domains of community empowerment and corresponding activities identified by Rapla community members.

**Domain**	**Activities**
**Community Activation**	- Activities to support community members’ participation in community problem solving processes - Involvement and engagement of more stakeholders - Motivation of new leaders - Creation and encouragement of new networks - Initiation and stimulation of new community groups, *etc.*
**Community Competence**	- Training to improve awareness and knowledge of community members to solve community problems - Distribution of information on good practices and evidence-based approaches - Information sharing to improve understanding of concepts, determinants and theories in health promotion, *etc.*
**Program management skills**	- Teaching of program management and team building skills - Training for planning, implementation and evaluation techniques - Instruction about information use, dissemination and communication skills - Improving community groups, abilities and expertise in the use of evidence-based techniques in identifying, solving and managing their problems, *etc.*
**Creation of supportive environment**	- Training community members in lobbying skills - Advocating for political support and financial resources - Promoting better access to different foundations and expert resources - Improving participants’ abilities to maintain and sustain political changes and achieve large social support, *etc.*

ODCE: organizational domains of community empowerment.

**Table 3.** A fragment of the ODCE measurement tool.

**I domain: Community activation Level 1**	**Not at all/Very limited** **1**	**Somewhat** **2**	**Substantial** **3**	**Almost entirely/Entirely** **4**
1. There exists a group of community representatives that meets regularly to work on community goals and desired community outcomes.				
2. The community group has an active leader(s), who motivates and enthuses members of group.				
3. The community workgroup is committed to solving local problems and is motivated to collaborate as a team.				

I A community workgroup is constituted, which cares for community problems, have active leaders and is committed to collaborate in solving the community’s problems.				

Mean 2003 ........				
Mean 2004 ........				
Mean 2005 ........				

Evidence describing the above mentioned assertions:				
2003................................................................................................................................................................................				
2004................................................................................................................................................................................				
2005................................................................................................................................................................................				

**Table 4.** Issue-specific processes: some activities undertaken by community workgroups.

**Community Initiative**	**Issue-Specific Activities**
**Safe Community**	- Organizing safety campaigns - Teaching school-children traffic behaviour - Publishing printed materials for mothers of newborn babies on prevention of baby’s injuries - Organizing swimming courses to prevent drowning - Implementing safe school campaigns - Publishing printed materials for elderly persons in order to prevent falls - Distribution of grants to stimulate small prevention projects, *etc.*
**Drug Abuse and AIDS Prevention**	- Organizing educational courses for your people to increase awareness - Lobbying local policy makers to support regulation of the night sales of alcohol and to reduce youths’ access to alcohol - Organizing alternative activities for the youth (summer-camps, drug-free discos) - Implementing anti-AIDS campaign and distribution of condoms to young people - Producing printed material on sexual education for young people
**Elderly Quality of Life**	- Organizing physical activity events in nature and in sport-halls - Advocating policy makers to achieve social benefits for elderly in needs - Organizing capacity building trainings - Organizing picnics and cultural outings - Inviting experts to talk on and debate health issues - Undertaking social support visits to peers - Implementing elderly Health Days, *etc.*

**Table 5.** Distribution of the gender and age characteristics of the workshop participants.

**Community Initiative**	**Safe Community**			**Drug Abuse and AIDS Prevention**			**Elderly Quality of Life**		

**Year**	**2003**	**2004**	**2005**	**2003**	**2004**	**2005**	**2003**	**2004**	**2005**
Male (N)	7	8	8	8	8	8	0	0	0
Female (N)	9	12	9	6	6	7	15	18	17
Total (N)	16	20	17	14	14	15	15	18	17
Age range (years)	29–68	30–69	31–69	24–52	25–53	26–54	48–72	49–73	49–74
Mean age (years)	42.5	44.8	44.1	32.4	32.4	36.1	62.2	62.8	63.4

**Table 6.** Community empowerment domains assessed by the *Safe Community* program workgroup.

**Domain**	**Year 2003** **N = 16**		**Year 2004** **N = 20**		**Year 2005** **N = 17**	

	**Mean**	**Range**	**Mean**	**Range**	**Mean**	**Range**
Activation of the community	1.96	1.3–2.6	2.53	2.0–3.3	3.20	2.6–4.0
Competence of the community	1.20	1.0–1.3	1.96	1.6–2.3	2.60	2.6–2.6
Program management skills	1.30	1.0–1.6	2.06	1.3–2.6	2.76	2.3–3.0
Creation of a supportive environment	1.13	1.0–1.3	1.63	1.3–2.0	2.30	2.0–2.6

**Table 7.** Community empowerment domains assessed by the *Drug Abuse and AIDS Prevention* program workgroup.

**Domain**	**Year 2003** **N = 14**		**Year 2004** **N = 14**		**Year 2005** **N = 15**	

	**Mean**	**Range**	**Mean**	**Range**	**Mean**	**Range**
Activation of the community	1.63	1.0–2.6	2.53	2.0–3.3	3.20	2.6–4.0
Competence of the community	1.20	1.0–1.3	1.96	1.6–2.3	2.6	2.6–2.6
Program management skills	1.30	1.0–1.6	2.06	1.3–2.6	2.76	2.3–3.0
Creation of a supportive environment	1.10	1.0–1.3	1.96	1.3–2.0	2.30	2.0–2.6

**Table 8.** Community empowerment domains assessed by the *Elderly Quality of Life* program workgroup.

**Domain**	**Year 2003** **N = 15**		**Year 2004** **N = 18**		**Year 2005** **N = 17**	

	**Mean**	**Range**	**Mean**	**Range**	**Mean**	**Range**
Activation of the community	1.63	1.3–2.0	2.43	2.0–3.0	2.73	2.6–3.0
Competence of the community	1.66	1.0–2.0	2.20	2.0–2.3	2.30	2.0–2.6
Program management skills	1.10	1.0–1.3	1.96	1.6–2.3	2.30	2.3–3.6
Creation of a supportive environment	1.63	1.0–2.6	2.16	1.3–2.6	1.63	1.3–2.0
